# Selective cyclooxygenase inhibition by SC-560 improves hepatopulmonary syndrome in cirrhotic rats

**DOI:** 10.1371/journal.pone.0179809

**Published:** 2017-06-20

**Authors:** Ching-Chih Chang, Wen-Shin Lee, Hsian-Guey Hsieh, Chiao-Lin Chuang, Hui-Chun Huang, Fa-Yauh Lee, Shou-Dong Lee

**Affiliations:** 1 Division of General Medicine, Department of Medicine, Taipei Veterans General Hospital, Taipei, Taiwan; 2 National Yang-Ming University School of Medicine, Taipei, Taiwan; 3 Department of Medical Research, Taipei Veterans General Hospital, Taipei, Taiwan; 4 Division of Gastroenterology, Department of Medicine, Taipei Veterans General Hospital, Taipei, Taiwan; 5 Cheng-Hsin General Hospital, Taipei, Taiwan; IDIBAPS Biomedical Research Institute, SPAIN

## Abstract

**Objective:**

Hepatopulmonary syndrome (HPS) is characterized by hypoxia in patients with chronic liver disease. The mechanism of HPS includes pulmonary vasodilatation, inflammation, and angiogenesis. Prostaglandins synthesized by cyclooxygenases (COX) participate in vascular responsiveness, inflammation and angiogenesis, which can be modulated by COX inhibitors. We therefore evaluated the impact of COX inhibition in rats with common bile duct ligation (CBDL)-induced liver cirrhosis and HPS.

**Methods:**

Cirrhotic rats were randomly allocated to receive non-selective COX inhibitor (indomethacin), selective COX-1 inhibitor (SC-560), or COX-2 inhibitor (celecoxib) for 14 days. After that, hemodynamic parameters, severity of hypoxia and intrapulmonary shunts, liver and renal biochemistry parameters, histological finding and protein expressions were evaluated.

**Results:**

Non-selective COX inhibition by indomethacin improved hepatic fibrosis and pulmonary inflammation in cirrhotic rats with HPS. It also decreased mean arterial blood pressure, portal pressure, and alleviated hypoxia and intrapulmonary shunts. However, indomethacin increased mortality rate. In contrast, selective COX inhibitors neither affected hemodynamics nor increased mortality rate. Hypoxia was improved by SC-560 and celecoxib. In addition, SC-560 decreased intrapulmonary shunts, attenuated pulmonary inflammation and angiogenesis through down-regulating COX-, NFκB- and VEGF-mediated pathways.

**Conclusion:**

Selective COX-1 inhibitor ameliorated HPS by mitigating hypoxia and intrapulmonary shunts, which are related to anti-inflammation and anti-angiogenesis.

## Introduction

The hepatopulmonary syndrome (HPS) is a dreadful complication in patients with chronic liver disease [1]. Three important components of HPS have been identified, including hypoxia with elevation of alveolar arterial oxygen pressure gradient (AaPO_2_), intrapulmonary vasodilatation with increased intrapulmonary shunts, and chronic liver disease [1,2]. The intrapulmonary vasodilatation and increased shunts lead to abnormal gas exchange and hypoxia in HPS patients [2]. Emerging studies showed that modulations of intrapulmonary inflammation and angiogenesis could reverse HPS in cirrhotic rats [3,4].

Cirrhosis of liver causes portal hypertension. As the portal pressure (PP) elevates, portal-systemic collaterals develop gradually to diverse excessive blood flow from the portal system [5]. Prostacyclin is a vasodilatory prostanoid which is capable of increasing portal tributary blood flow and portal pressure [6]. It is synthesized through cyclooxygenases (COX) including COX-1 and COX-2. The previous studies have reported that COX inhibition attenuated collateral vasodilatation in portal hypertensive and cirrhotic rats [7,8]. In addition, accumulating evidences have identified that COX inhibitors ameliorated acute and chronic liver injury, liver fibrosis and steatosis, abnormal vascular responsiveness, and excessive angiogenesis [9–11]. However, the impact of COX inhibition on HPS is still lacking. In order to address this issue, in this study, rats with common bile duct ligation (CBDL)-induced liver cirrhosis and HPS were used [3,4]. The non-selective COX inhibitor (indomethacin), selective COX-1 inhibitor (5-(4-chlorophenyl)-1- (4-methoxyphenyl)-3-trifluoromethyl pyrazole, SC-560), and COX-2 inhibitor (celecoxib) were administered to evaluate their influences on HPS.

## Materials and methods

### Animal model

Male Sprague-Dawley rats weighing 240–270 g at the time of surgery were used for experiments. Under ketamine anesthesia (100 mg/kg, intramuscularly), CBDL was performed and a high yield of secondary biliary cirrhosis was noted after four weeks of CBDL [12,13]. To avoid the coagulation defect, CBDL rats received weekly vitamin K injection (50 μg/kg intramuscularly). In the survival study, we anesthetized the rats with ketamine injection before and during operation until they recovered. During the experimental period, we monitored the rats every day to calculate the survival rate. On the day of experiment, the rats were under anesthesia throughout the whole course of experiment then euthanasia was applied. At the end of experiment, we used potassium chloride injection intravenously to euthanize experimental animal. All the experiments were designed to be adhered to the American physiological society guiding principles for the care and use of laboratory animals (NIH publication no. 86–23, revised 1985). This study was approved by the Taipei Veterans General Hospital Animal Committee (IACUC 2011–191).

### Study protocol

#### The 1^st^ series

CBDL rats were intraperitoneally injected with indomethacin (5 mg/kg/day) or vehicle (0.9% sodium chloride 1 ml/day, control group) from the 14^th^ to 28^th^ day post CBDL. On the 28^th^ day after CBDL, the mortality rate and hemodynamic data were measured. Blood was collected for the measurement of liver and renal biochemistry data [alanine transaminase (ALT), aspartate aminotransferase (AST), total bilirubin and creatinine] and blood gas analysis. On the paralleled groups, intrapulmonary shunts were determined using color microsphere technique. Furthermore, liver and lung were dissected for histopathological examinations.

#### The 2^nd^ series

The selective COX-1 inhibitor (SC-560, 3mg/kg/day), COX-2 inhibitor (celecoxib, 20mg/kg/day), or vehicle (1 ml DMSO, control group) were intraperitoneally administered to the CBDL rats from the 14^th^ to 28^th^ day post operation. The mortality rate, hemodynamic data, plasma concentrations of liver and renal biochemistry parameters, tumor necrosis factor alpha (TNF-α) and interleukin-1β (IL-1β) were measured and blood gas analysis was performed. Intrapulmonary shunts were determined in paralleled groups. Lungs and livers were dissected for histopathological examinations, protein analyses and immunohistochemical staining.

### Systemic and portal hemodynamic measurements

The right internal carotid artery was cannulated with a PE-50 catheter for continuous recordings of mean arterial pressure (MAP) and heart rate (HR) on a multi-channel recorder (model RS 3400, Gould Inc., Cupertino, CA, USA). The external zero reference was placed at the level of the mid-portion of the rat. The abdomen was opened and a mesenteric vein was cannulated with a PE-50 catheter to measure the PP.

### Determination of plasma TNF-α and IL-1β levels

The plasma levels of TNF-α and IL-1β were measured by using commercially available enzyme-linked immunoabsorbent assay kits (R&D Systems, Inc., Minneapolis, MN, USA) according to the manufacturer’s instructions.

### Western analysis for protein expressions

The protein extracts of lung were incubated with the primary antibody [anti-phosphoinositide 3-kinases (PI3K) (1:1000; Cell Signaling Technology); anti-nuclear factor kappa B (NFκB, p65) (1:200; Santa Cruz Biotechnology, Santa Cruz, CA, U.S.A.); anti-nuclear factor of kappa light polypeptide gene enhancer in B-cells inhibitor alpha (IκBα) (1:10000; Abcam plc); anti-phosphorylated IκBα (1:1000; Cell Signaling Technology); anti-VEGF (1:1000; Santa Cruz Biotechnology); anti-VEGFR-1, -phosphorylated VEGFR-1 (1:1000; Abcam plc); anti-VEGFR-2 (1:500; Millipore Corporation); anti-phosphorylated VEGFR-2 (1:1000; Cell Signaling Technology); anti-Rho-associated kinase (RhoA) (1:1000; Cell Signaling Technology); anti-Akt (1:500, Cell Signaling Technology); anti-phosphorylated Akt (1:2000, Cell Signaling Technology); anti-extracellular signal-regulated kinase (ERK), -phosphorylated ERK (1:3000, Millipore Corporation)]. Then the blots were incubated with the secondary antibody (horseradish peroxidase-conjugated goat anti-mouse IgG antibody, Sigma Chemical Co., St. Louis, MO, U.S.A.). With a computer assisted video densitometer and digitalized software (Kodak Digital Science^TM^ ID Image Analysis Software, Eastman Kodak Co., Rochester, NY, U.S.A.), the blots were scanned, photographed then the signal intensity (integral volume) of the appropriate bands were analyzed.

### Intrapulmonary shunting analysis

Intrapulmonary shunts were determined by a microsphere method [14,15]. In brief, the femoral artery and vein were cannulated with PE-50 catheters, one day prior to the experiments. The cross-linked (2.5 x 10^6^) colored microspheres (size range 6.5–10 μm; Interactive Medical Technologies, Los Angeles, CA, U.S.A.) were injected through the femoral vein, which were immediately flushed with 0.2 ml of sterile normal saline. A reference blood sample was withdrawn from the femoral artery at the time of femoral vein injection for a total of 90 seconds at a constant rate of 1.0 ml/min. The numbers of colored microspheres beads were coded using a hemacytometer counting slide having a known cell volume. Total numbers of microspheres passing through the pulmonary microcirculation were calculated as (reference blood sample microspheres/ml) × estimated blood volume. The estimated blood volume (ml) of each animal was 0.06 x body wt (g) + 0.77 [16]. Intrapulmonary shunting degree was calculated as an intrapulmonary shunt fraction (%): (total numbers of microspheres passing through the pulmonary microcirculation/total beads injected into the venous circulation) x 100.

### Histopathological and immunohistochemical staining

The liver and lung were dissected free and fixed in 10% formalin solution. The sections were stained with Hematoxylin and Eosin (H&E) and examined by light microscopy. Liver sections were stained with Sirius Red (Polysciences Inc., Warrington, PA, U.S.A.) to determine the extent of collagen deposition. The immunohistochemical staining was performed with anti-CD68 antibody (diluted 1:200, ab31630, Abcam Cambridge, UK) to detect pulmonary CD68-positive macrophages, with anti-CD31 antibody (1:200; Serotec and Pharmingen, San Diego, CA) to determine hepatic angiogenesis, and with anti-von Willebrand factor (vWF) antibody (1:100, MCA127T, AbD Serotec, UK) to determine pulmonary angiogenesis [4,17]. The numbers of CD68-, CD31-, and vWF-positive cells per high-power field (magnification 200x) were counted by a semi-quantification method [18].

### Drugs

Indomethacin and SC-560 were purchased from Sigma Chemical Co. (St. Louis, MO. U.S.A.). Celecoxib was purchased from Pfizer Pharmaceuticals (Pfier Inc., NY, U.S.A.). All the solutions were freshly prepared on the days of experiments.

### Data analysis

The results are expressed as mean ± standard deviation. Statistical analyses were performed by one-way analysis of variance test with post hoc test by Tukey analysis or Student’s t test when appropriate. The survival was analyzed with Log-rank test and survival curve drawn with Kaplan-Meier graph. Results were considered statistically significant at a P value less than 0.05.

## Results

### Mortality and morbidity

**Fig 1:**
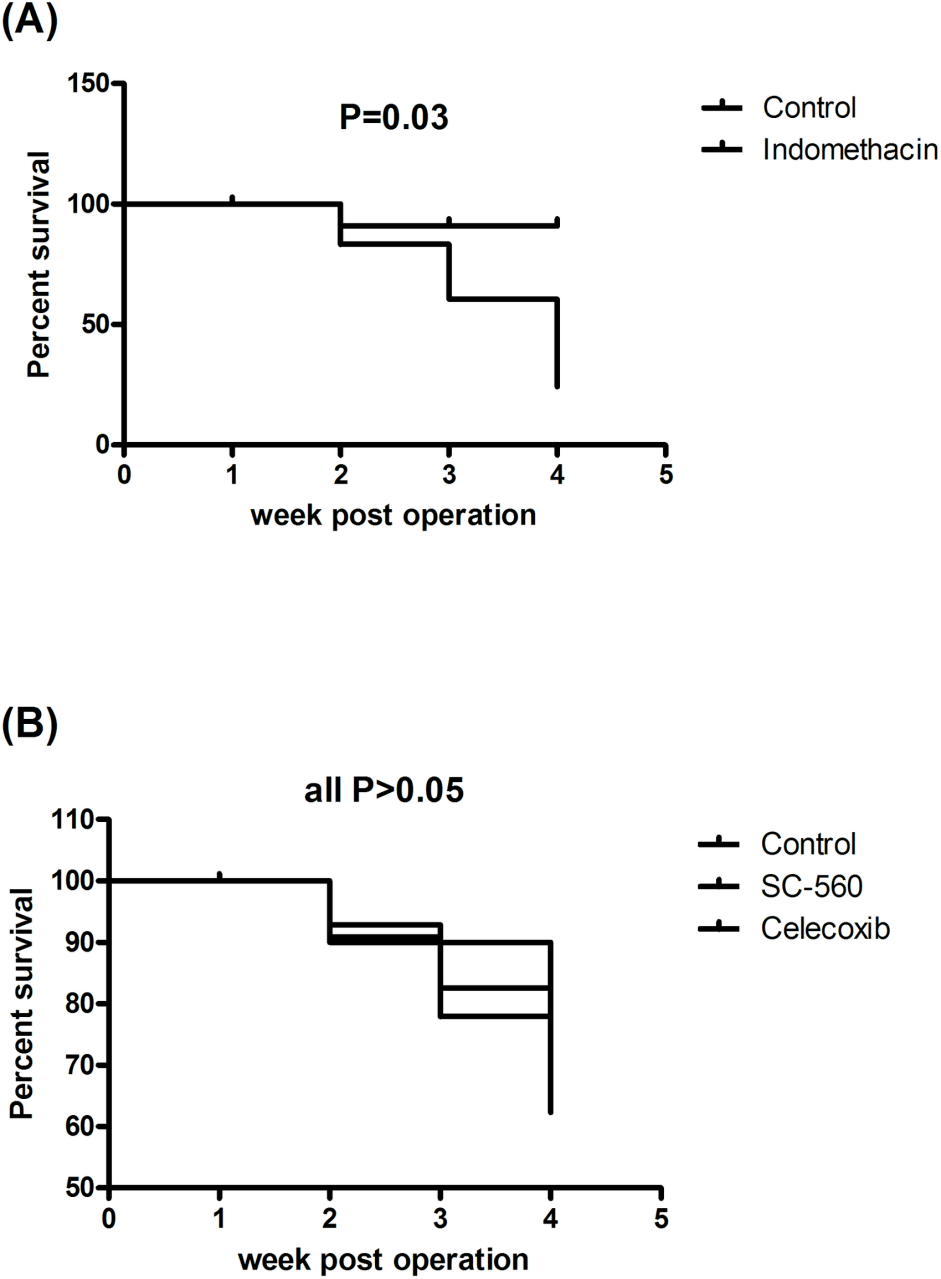
Survival curve (Kaplan-Meier graph) in cirrhotic rats with or without COX-inhibitor treatment. Indomethacin significantly decreased the survival rate as compared with the control group (A). There was no significant difference among SC-560, celecoxib and control groups (B).

Indomethacin significantly increased the mortality rate in CBDL rats [indomethacin: control = 36% (9 of the 25 rats died) vs. 6.6% (1 of the 15 rats died), P = 0.03]. Most of the indomethacin-treated rats died during the 2^nd^ week of indomethacin treatment (Fig 1A). Neither SC-560 nor celecoxib increased the mortality rate [SC-560: control: celecoxib = 15.4% (2 of the 13 rats died) vs. 18.7% (3 of the 16 rats died) vs. 11.1% (2 of the 18 rats died), P ≥ 0.05 among groups, Fig 1B]. Rats with or without COX inhibitors treatment developed significant jaundice and ascites formation, four weeks post CBDL operation.

### Hemodynamics, biochemistries and intrapulmonary shunts of control and indomethacin-treated groups

Table 1 depicts the hemodynamics, biochemistry data and pulmonary shunting degree of indomethacin (n = 10) and control (vehicle, n = 9) groups. As compared with the control group, indomethacin significantly decreased MAP (mmHg) and PP (mmHg) of CBDL rats (indomethacin vs. control: MAP: 92 ± 10 vs. 104 ± 10; PP: 14.3 ± 3.0 vs. 17.8 ± 2.7, both P < 0.05) without influencing heart rate. Indomethacin also reduced the total bilirubin (mg/dL) level (5.5 ± 1.5 vs. 7.7 ± 0.5, P < 0.05) without affecting the levels of ALT, AST, and creatinine. Besides, indomethacin decreased intrapulmonary shunts (%) (9.0 ± 5.3 vs. 22.2 ± 8.6, P < 0.05) and AaPO_2_ (mmHg) (11.0 ± 4.5 vs. 16.2 ± 2.5, P < 0.05).

**Table 1:** Hemodynamic, biochemistry parameters, intrapulmonary shunts and blood gas analysis of CBDL rats receiving indomethacin or vehicle (control) treatment.

	Indomethacin (n = 10)	Control (n = 9)
Body weight (g)	363 ± 26	373 ± 20
MAP (mmHg)	92 ± 10*	104 ± 10
PP (mmHg)	14.3 ± 3.0*	17.8 ± 2.7
HR (beats/min)	356 ± 25	376 ± 19
ALT (IU/L)	156 ± 61	184 ± 48
AST (IU/L)	984 ± 305	1074 ± 210
TB (mg/dL)	5.5 ± 1.5*	7.7 ± 0.5
Cr (mg/dL)	0.2 ± 0.09	0.2 ± 0.04
Shunt degree (%)	9.0 ± 5.3*	22.2 ± 8.6
PaO_2_ (mmHg)	92.7 ± 4.9	89.1 ± 4.0
PaCO_2_ (mmHg)	37.0 ± 2.8	35.8 ± 2.3
AaPO_2_ (mmHg)	11.0 ± 4.5*	16.2 ± 2.5

MAP: mean arterial pressure; PP: portal pressure; HR: heart rate; AST: aspartate aminotransferase; ALT: alanine aminotransferase; TB: total bilirubin; Cr: creatinine; PaO_2_: partial pressure of oxygen; PaCO_2_: partial pressure of carbon dioxide; AaPO_2_: alveolar arterial oxygen pressure gradient

* P < 0.05 between indomethacin-treated and control group.

### Hemodynamics, biochemistries, plasma levels of TNF-α, IL-1β and intrapulmonary shunts in SC-560- and celecoxib-treated groups

Table 2 reveals the hemodynamics, biochemistry data, plasma levels of TNF-α, IL-1β and intrapulmonary shunts in celecoxib (n = 10), control (vehicle, n = 9) and SC-560 (n = 7) groups. As compared with vehicle, neither selective COX-1 nor COX-2 inhibition affected the hemodynamics and liver and renal biochemistries. Selective COX inhibitions significantly decreased the plasma levels of TNF-α and IL-1β (pg/mL) (celecoxib vs. control vs. SC-560: TNF-α = 20.8 ± 7.8 vs. 32.2 ± 8.7 vs. 19.8 ± 3.8, celecoxib and SC-560 vs. control, both P < 0.05; IL-1β: 65.5 ± 25.2 vs. 110.9 ± 40.3 vs. 67.2 ± 26.7, celecoxib and SC-560 vs. control, both P < 0.05). SC-560 significantly reduced the intrapulmonary shunts (%) (SC-560 vs. control: 9.6 ± 2.6 vs. 17.5 ± 1.9%, P < 0.05), which was not found in celecoxib group (celecoxib vs. control: 17.7 ± 1.4 vs. 17.5 ± 1.9%, P ≥ 0.05). In addition, SC-560 significantly increased the partial pressure of oxygen (PaO_2_, mmHg) and decreased AaPO_2_ (mmHg) (SC-560 vs. control: PaO_2_: 92.9 ± 5.1 vs. 86.6 ± 3.7; AaPO_2_: 13.6 ± 3.7 vs. 18.0 ± 3.9, both P < 0.05). Celecoxib exerted the same significant effects (celecoxib vs. control: PaO_2_: 92.2 ± 3.0 vs. 86.6 ± 3.7; AaPO_2_: 14.0 ± 3.1 vs. 18.0 ± 3.9, both P < 0.05). The PaO_2_ and AaPO_2_ were not significantly different between SC-560- and celecoxib-treated CBDL rats.

**Table 2:** Hemodynamics, biochemistry, TNF-α, IL-1β, intrapulmonary shunts and blood gas analysis of CBDL rats receiving celecoxib, vehicle (control) or SC-560 treatment.

	Celecoxib(n = 10)	Control (n = 9)	SC-560 (n = 7)
Body weight (g)	370 ± 23	353 ± 28	369 ± 43
MAP (mmHg)	106 ± 12	106 ± 16	117 ± 14
PP (mmHg)	17.9 ± 4.6	17.2 ± 2.9	17.2 ± 4.4
HR (beats/min)	375 ± 25	357 ± 51	394 ± 44
ALT (IU/L)	165 ± 33	194 ± 124	206 ± 134
AST (IU/L)	1034 ± 256	1201 ± 637	1462 ± 902
TB (mg/dL)	7.8 ± 1.0	9.3 ± 2.4	7.8 ± 0.3
Cr (mg/dL)	0.18 ± 0.01	0.19 ± 0.04	0.17 ± 0.02
TNF-α (pg/mL)	20.8 ± 7.8*	32.2 ± 8.7	19.8 ± 3.8*
IL-1β (pg/mL)	65.5 ± 25.2*	110.9 ± 40.3	67.2 ± 26.7*
Shunt degrees (%)	17.7 ± 1.4	17.5 ± 1.9	9.6 ± 2.6*
PaO_2_ (mmHg)	92.2 ± 3.0*	86.6 ± 3.7	92.9 ± 5.1*
PaCO_2_ (mmHg)	35.1 ± 2.1	36.4 ± 3.1	34.8 ± 3.7
AaPO_2_ (mmHg)	14.0 ± 3.1*	18.0 ± 3.9	13.6 ± 3.7*

MAP: mean arterial pressure; PP: portal pressure; HR: heart rate; AST: aspartate aminotransferase; ALT: alanine aminotransferase; TB: total bilirubin; Cr: creatinine; TNF-α: tumor necrosis factor-α; IL-1β: interleukin-1β; PaO_2_: partial pressure of oxygen; PaCO_2_: partial pressure of carbon dioxide; AaPO_2_: alveolar arterial oxygen pressure gradient

* P < 0.05 compared to control group.

### Effects of indomethacin on the histopathological change

**Fig 2:**
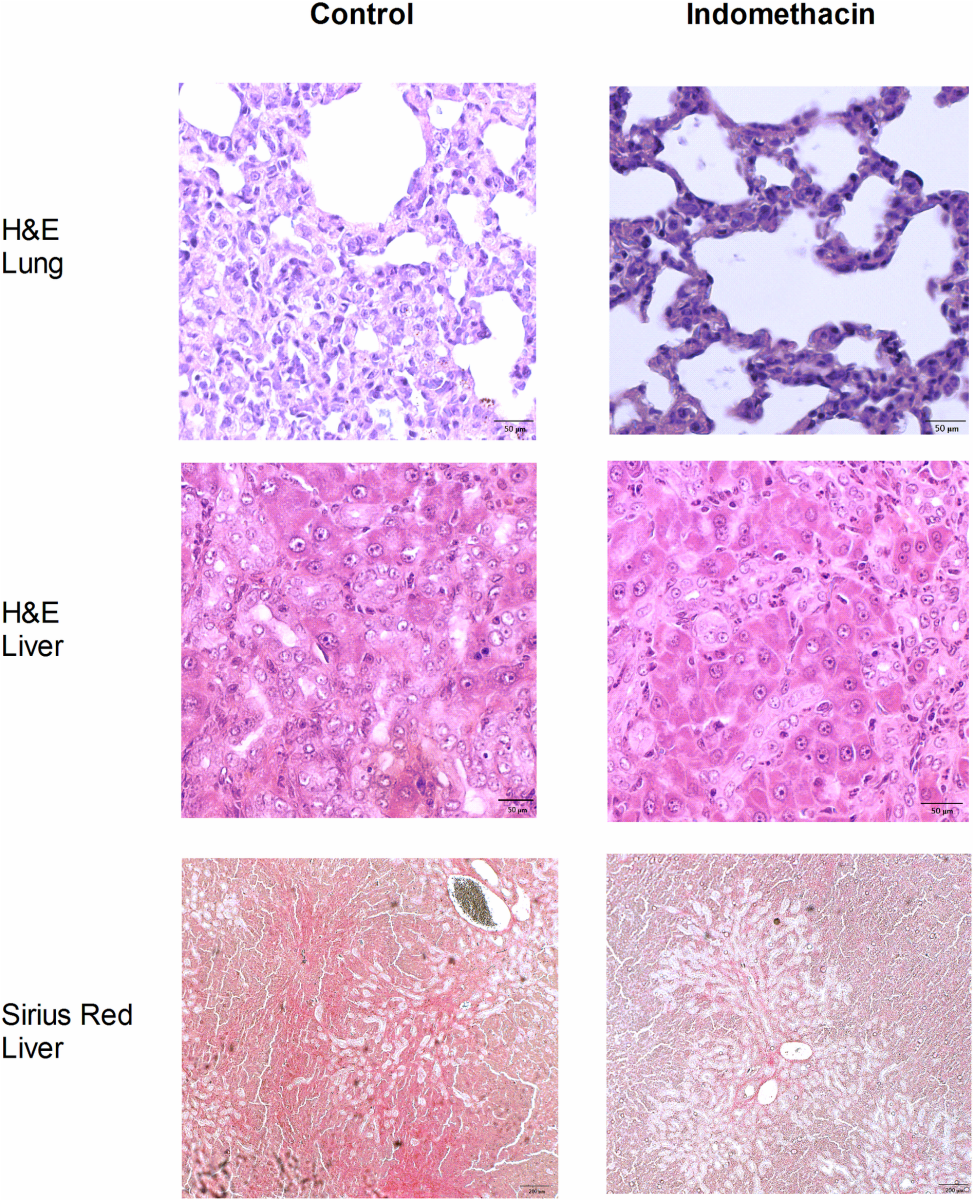
Pulmonary and hepatic histology of CBDL rats with or without indomethacin treatment. The representative pulmonary H&E staining showed prominant polymorphonuclear cells accumulation with tissue necrosis and disruption of normal alveoli (magnification 200x). The infiltrative cells were reduced by indomethacin. Hepatic H&E staining revealed tissue necrosis and bile ductule proliferation, which were not influenced by indomethacin (magnification 200x). Hepatic Sirius Red staining revealed collagen fiber deposition (red in color), which was ameliorated by indomethacin (magnification 40x).

Fig 2 shows the histological changes induced by indomethacin. The intrapulmonary polymorphonuclear cells accumulation was significantly alleviated by indomethacin. The liver section of CBDL rats showed bile ductules proliferation and destruction of lobular structure, which were not influenced by indomethacin. Sirius Red staining revealed that indomethacin attenuated collagen fiber deposition, indicating the improvement of liver fibrosis.

### Effects of selective COX inhibitors on the pulmonary inflammation and angiogenesis

**Fig 3:**
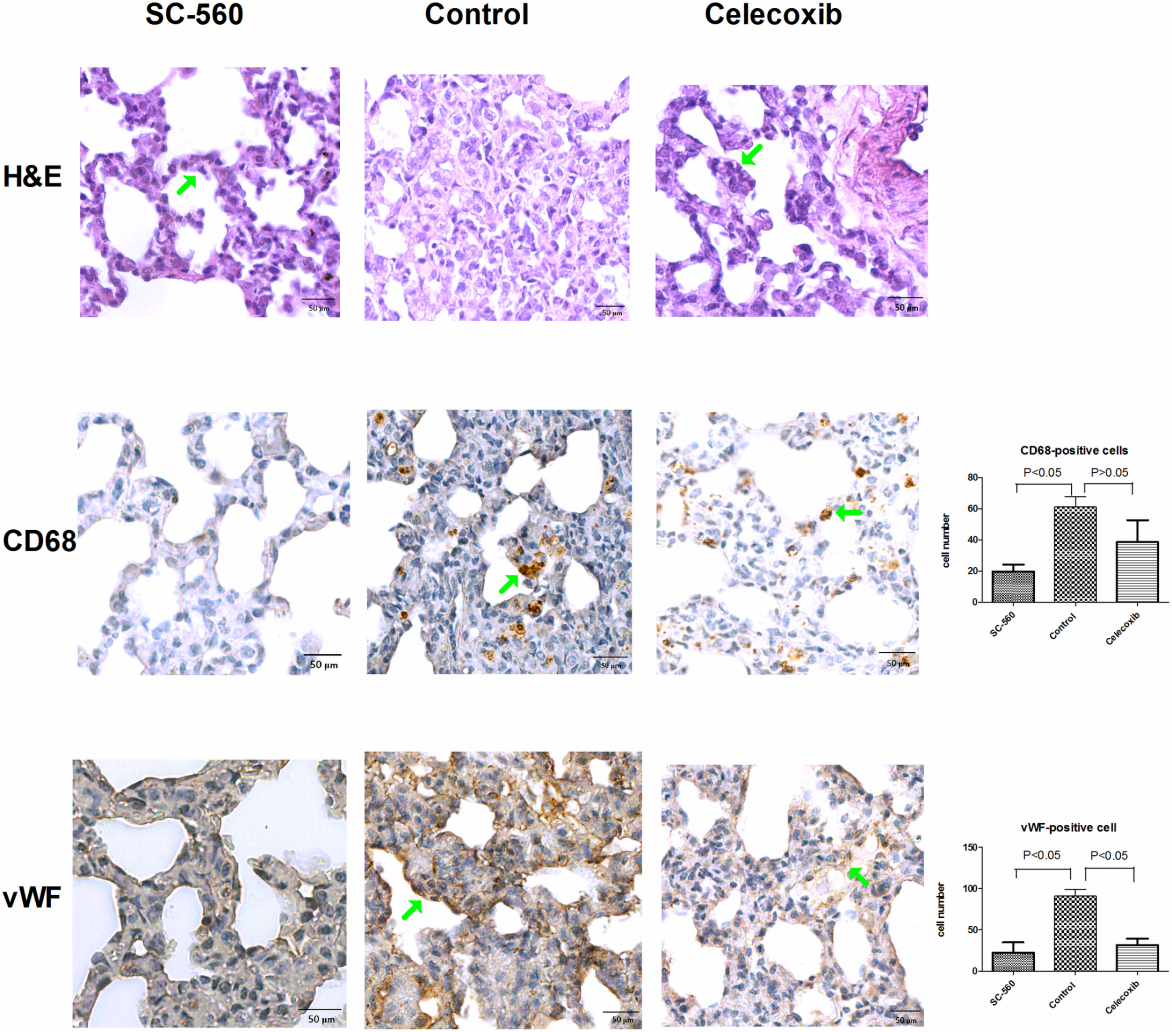
Pulmonary H&E and immunochemical staining in rats with selective COX inhibition. The representative H&E staining image showed polymorphonuclear cells infiltration around the alveoli (green arrow), which was ameliorated by SC-560 and celecoxib (magnification 200x). SC-560 diminished CD-68 positive macrophages (green arrow indicating brown cells) accumulation compared to control and celecoxib groups (magnification 200x). The pulmonary vWF-positive cells (green arrow indicating brown cells) were decreased by SC-560 and celecoxib, as compared to control group (magnification 200x). The numbers of CD-68 positive and vWF-positive cells in the high power field are shown in the right lower corner.

Fig 3 reveals the effects of selective COX inhibitors on the pulmonary inflammation and angiogenesis. The pulmonary polymorphonuclear cells infiltration was ameliorated by SC-560 and celecoxib compared to vehicle. The lungs of CBDL rats have prominent CD68-positive staining macrophages infiltration, which was attenuated by SC-560 but not celecoxib (counts/per high power field) (SC-560 vs. control: 20 ± 8 vs. 61 ± 11; P < 0.05; celecoxib vs. control: 39 ± 24 vs. 61 ± 11; P ≥ 0.05). The vWF-positive staining cells were less in the SC-560- and celecoxib-treated rats compared to the vehicle-treated control rats (counts/per high power field) (celecoxib vs. control vs. SC-560: 32 ± 14 vs. 91 ± 14 vs. 22 ± 22; celecoxib and SC-560 vs. control, both P < 0.05).

### Effects of selective COX inhibitors on the intrahepatic inflammation, fibrosis and angiogenesis

**Fig 4:**
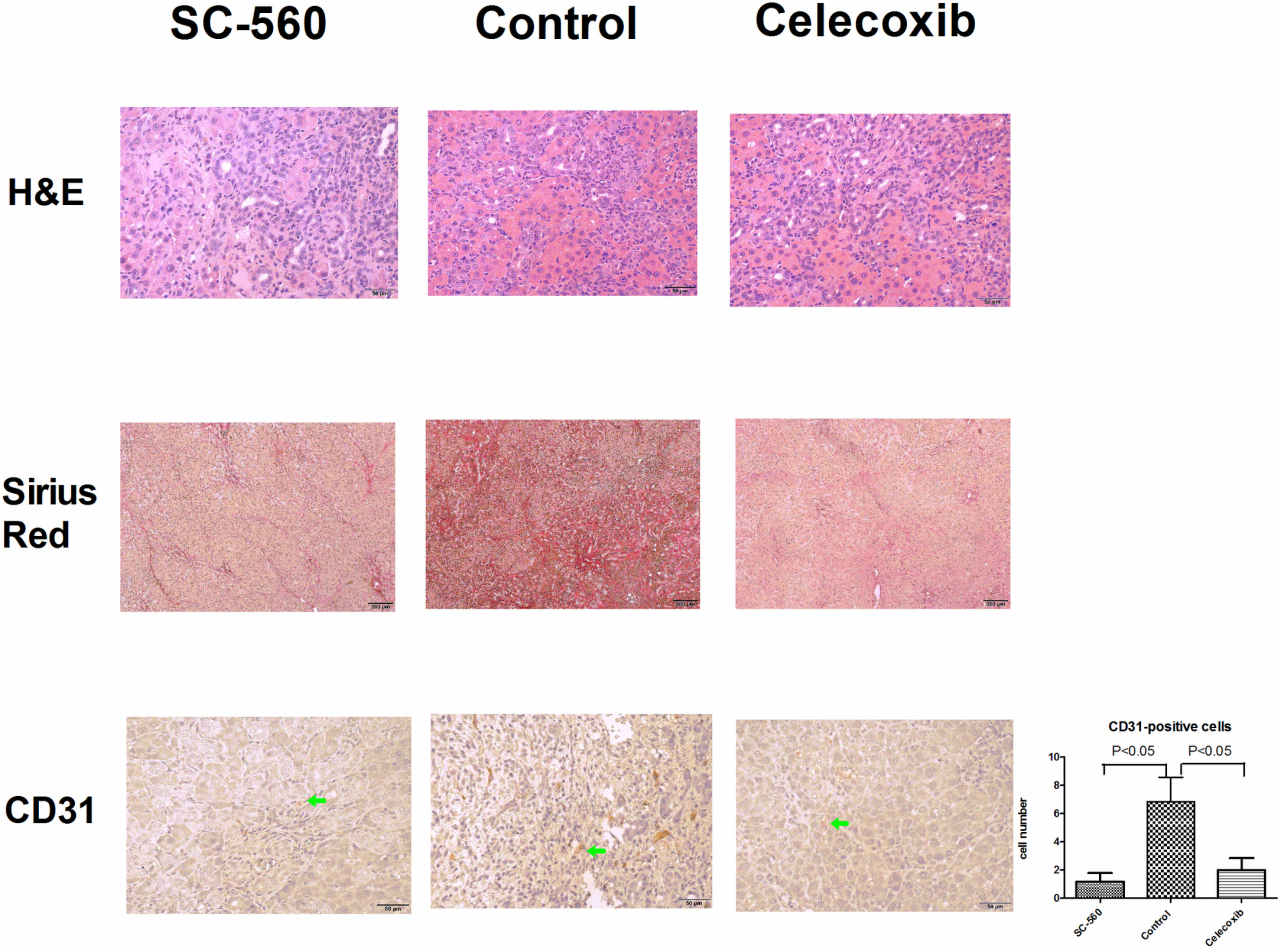
The liver histology of CBDL rats with or without selective COX-inhibitor treatments. The representative H&E staining showed hepatic tissue necrosis and bile ductule proliferation, which were not ameliorated by selective COX inhibitors (magnification 200x). Celecoxib and SC-560 significantly reduced CD31-positive cell numbers (brown cells, magnification 200x). The numbers of CD-31 positive cells in the high power field are shown in the right lower corner. Sirius Red staining revealed collagen fiber deposition (red in color), which was ameliorated by SC-560 and celecoxib (magnification 40x).

Fig 4 reveals the effects of selective COX inhibitors on the hepatic inflammation, fibrosis and angiogenesis. The intrahepatic polymorphonuclear cells infiltration was similar among SC-560-, celecoxib-treated and control groups. The liver fibrosis was attenuated by SC-560 and celecoxib as evidenced by Sirius Red staining. The CD31-positive staining cells were less in the SC-560- and celecoxib-treated rats compared to control rats (counts/per high power field) (celecoxib vs. control vs. SC-560: 2 ± 2 vs. 7 ± 4 vs. 1 ± 1; celecoxib and SC-560 vs. control, both P < 0.05).

### The pulmonary protein expressions of CBDL rats treated by selective COX inhibitors

**Fig 5:**
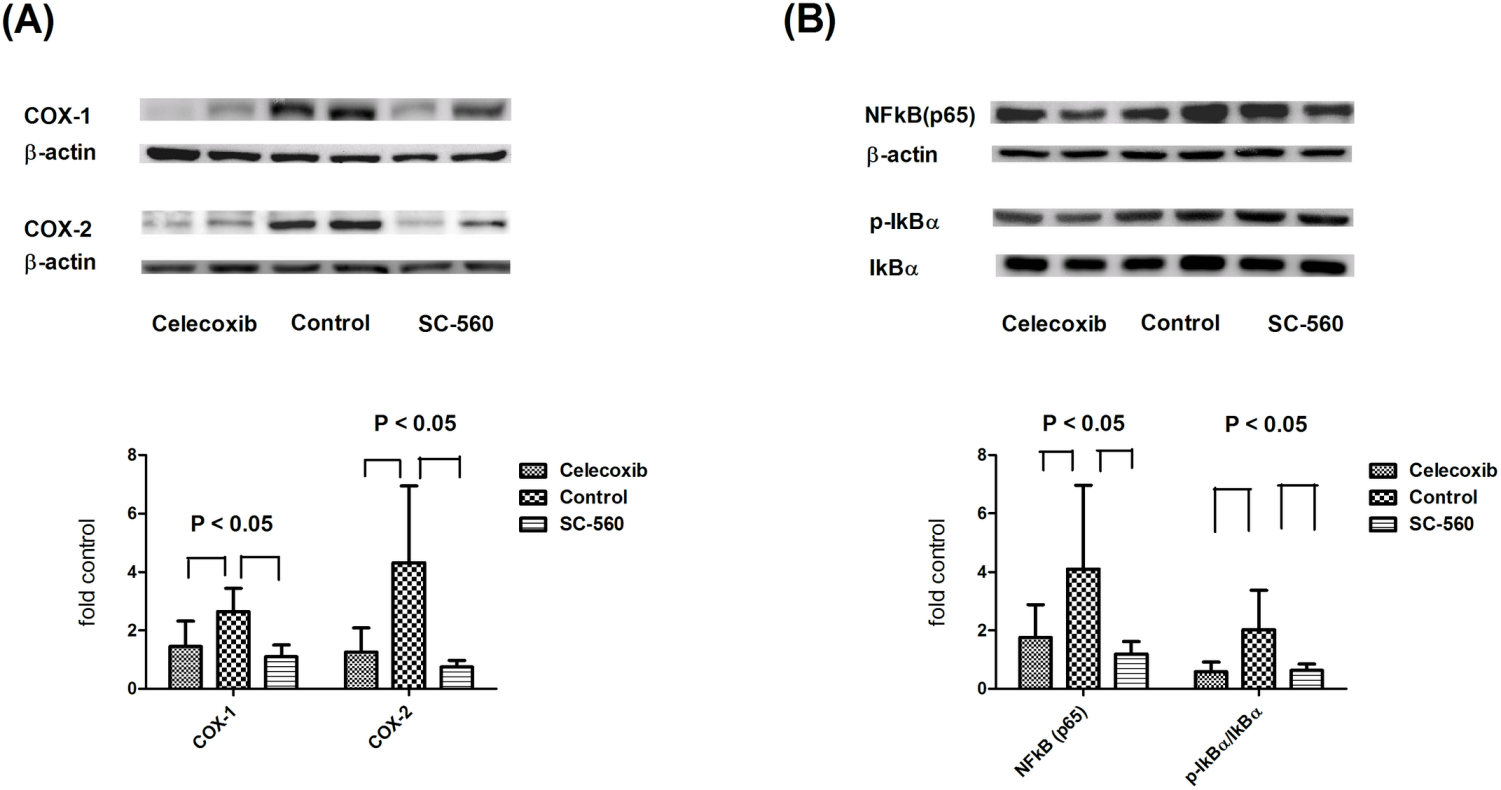
Pulmonary protein expressions of COX-1, COX-2, NFκB and IκBα. COX-1 and COX-2 protein expressions were down-regulated by SC-560 and celecoxib treatments (5A). NFκB (p65) and phosphorylated IκBα expressions were also significantly down-regulated by SC-560 and celecoxib treatments (5B).

**Fig 6:**
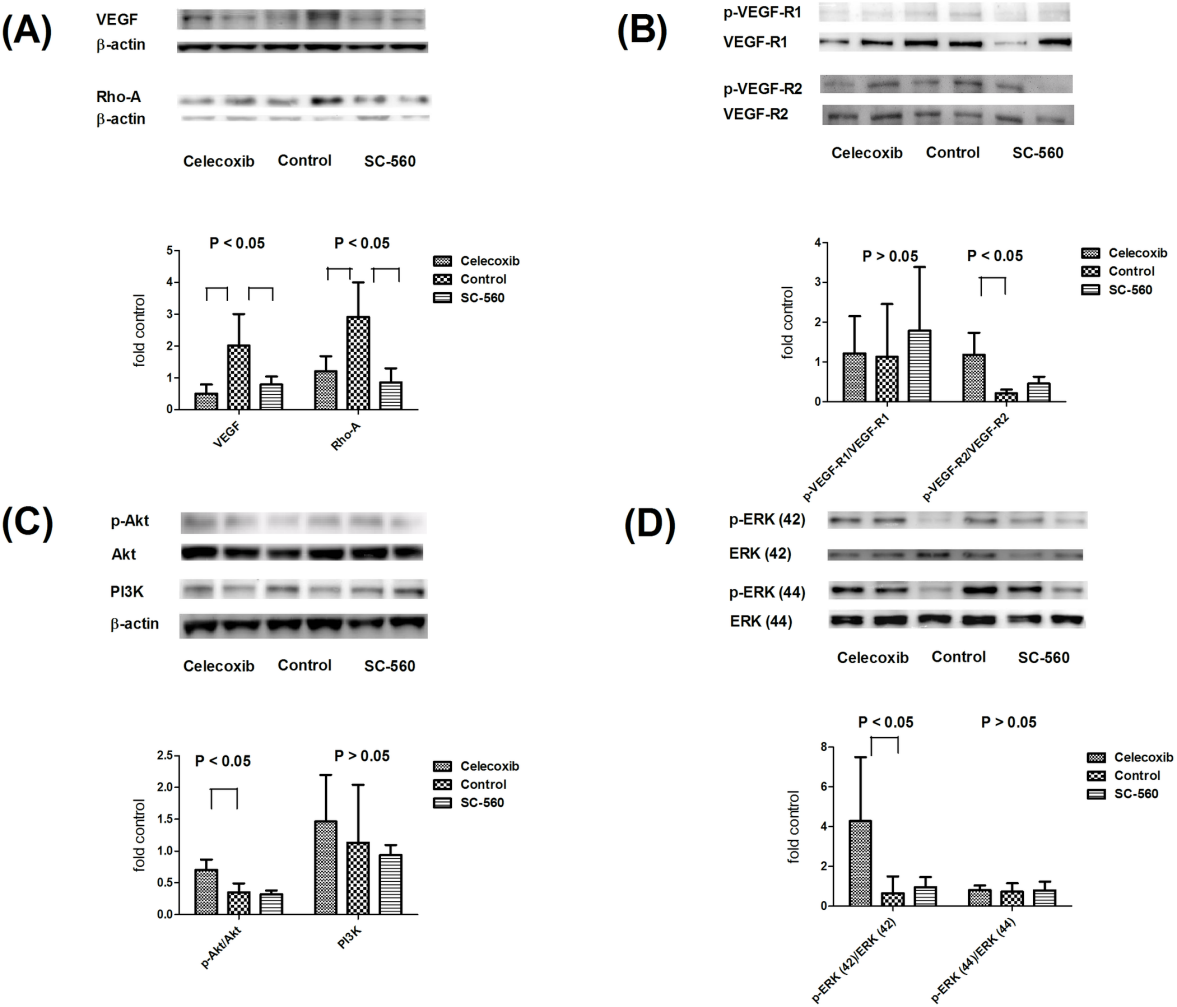
Pulmonary protein expressions of VEGF, RhoA, VEGFR-1, VEGFR-2, Akt, PI3K and ERK. SC-560 and celecoxib significantly down-regulated VEGF and RhoA expressions (6A). The phosphorylated VEGFR-2 expression was significantly up-regulated by celecoxib (6B). The phosphorylated Akt and ERK (42) protein levels were also significantly up-regulated by celecoxib (6C&D). The PI3K and phosphorylated ERK (44) expressions were not significantly influenced by celecoxib (6C&D).

Figs 5 and 6 show the pulmonary protein expressions of CBDL rats treated by selective COX inhibitors. COX-1 and COX-2 protein expressions were down-regulated by SC-560 and celecoxib [celecoxib vs. control vs. SC-560 (protein/ß-actin): COX-1 = 1.45 ± 0.87 vs. 2.64 ± 0.80 vs. 1.10 ± 0.41; COX-2 = 1.25 ± 0.80 vs. 4.32 ± 2.60 vs. 0.75 ± 0.23; celecoxib and SC-560 vs. control, both P < 0.05; celecoxib vs. SC-560, P ≥ 0.05; Fig 5A]. The NFκB (p65) and phosphorylated IκBα were also attenuated by selective COX inhibitors (NFκB = 1.76 ± 1.12 vs. 4.10 ± 2.87 vs. 1.19 ± 0.43; phosphorylated IκBα = 0.59 ± 0.32 vs. 2.02 ± 1.34 vs. 0.63 ± 0.21; celecoxib and SC-560 vs. control, both P < 0.05; Fig 5B).

The VEGF and RhoA protein expressions were also significantly down-regulated by SC-560 and celecoxib (celecoxib vs. control vs. SC-560: VEGF = 0.50 ± 0.29 vs. 2.01 ± 1.00 vs. 0.79 ± 0.25; RhoA = 1.20 ± 0.48 vs. 2.91 ± 1.09 vs. 0.85 ± 0.44; celecoxib and SC-560 vs. control, both P < 0.05; Fig 6A). The phosporylated VEGFR-1 protein expression was not significantly different among groups (1.22 ± 0.94 vs. 1.13 ± 1.32 vs. 1.79 ± 1.60; P ≥ 0.05; Fig 6B). However, celecoxib significantly elevated phosphorylated VEGFR-2 protein levels (1.18 ± 0.55 vs. 0.22 ± 0.09 vs. 0.45 ± 0.17; celecoxib vs. control, P < 0.05; Fig 6B). In addition, phosphorylated Akt and ERK (42) protein levels were up-regulated by celecoxib (phosphorylated Akt = 0.70 ± 0.16 vs. 0.35 ± 0.14 vs. 0.32 ± 0.06; celecoxib vs. control and SC-560, both P < 0.05; phosphorylated ERK (42) = 4.28 ± 3.21 vs. 0.64 ± 0.84 vs. 0.95 ± 0.50; celecoxib vs. control, P < 0.05; Fig 6C and 6D). The PI3K and phosphorylated ERK (44) were not influenced by selective COX inhibitions (PI3K = 1.47 ± 0.73 vs. 1.13 ± 0.91 vs. 0.94 ± 0.16; phosphorylated ERK (44) = 0.81 ± 0.21 vs. 0.73 ± 0.43 vs. 0.79 ± 0.44; P ≥ 0.05; Fig 6C and 6D).

## Discussion

The present study highlights that selective COX-1 inhibition improves HPS without detrimental effects on hemodynamics and biochemistries in an experimental animal model. Although cautions should be paid in extrapolating the findings of animal experiments to clinical practice, the clinical relevance is still worth noting: The control of HPS in cirrhotic patients has been extremely challenging, owing to the fact that apart from liver transplantation, there is no well documented agent recognized for HPS treatment [19]. Although further clinical investigation is required, our present finding suggests that the control of HPS might be achieved in a more accessible way without significant adverse impacts via selective COX inhibition. On the other hand, the non-selective COX inhibition by indomethacin also improves hypoxia and decreases the intrapulmonary shunts. However, it is not recommended in HPS treatment since indomethacin adversely influences hemodynamics and results in a higher mortality rate.

The effects of COX inhibition on systemic and hepatic hemodynamics have been investigated in the past but debates still exist. Bruix et al. found that a single dose of indomethacin significantly reduced hepatic blood flow and PP in cirrhotic patients [20]. In addition, acute and chronic COX blockades by indomethacin significantly decreased mesenteric arterial blood flow in portal hypertensive rats and reduced PP in portal hypertensive rabbits [21,22]. However, a contradictory report by Oberti et al. shows that acute administration of indomethacin did not affect cardiac output and PP in portal hypertensive and cirrhotic rats [6]. In our previous work, we found that 1-week indomethacin administration did not alter systemic hemodynamics, liver and renal biochemistry data and mortality rate in cirrhotic rats [23], S1 Table. However, the present study showed that a 2-week indomethacin administration significantly decreased MAP and PP of cirrhotic rats and increased the mortality rate without affecting liver and renal biochemistry parameters. In addition, most rats died during the 2^nd^ week of indomethacin treatment. It is thus inferred that the higher mortality rate of cirrhotic rats in the 2^nd^ week of indomethacin administration might be attributed, at least partially, to the systemic hypotension. Although indomethacin also improves HPS, the adverse hemodynamic and survival impacts preclude its use. On the contradictory, selective COX-1 and COX-2 inhibitors neither alter hemodynamics nor affect mortality, which are more promising than non-selective COX inhibition in HPS treatment.

The selective COX inhibitor has been proved to alleviate oxidative stress and inflammation in CCl_4_-treated rats [24]. Using TAA-induced cirrhotic rat model, Wen et al. have documented that selective COX-inhibition by celecoxib ameliorated hepatic fibrosis through amelioration of hepatic inflammation and angiogenesis [25]. We also found that selective COX-1 and COX-2 inhibition attenuated hepatic fibrosis and angiogenesis. However, PP was not influenced. The various findings can be ascribed to different experimental designs. Furthermore, the current study showed that selective COX-1 inhibition by SC-560 decreased the pulmonary CD68-positive macrophages and polymorphonuclear cells, indicating its anti-inflammatory effect. Selective COX inhibitors also reduced systemic levels of TNF-α and IL-1β, in which TNF-α participates in developing and maintaining HPS and TNF-α neutralization improves experimental HPS [2, 26]. Taken together, selective COX inhibition by SC-560 improves the systemic and pulmonary inflammation, which plays a role in the reversal of HPS.

In the present study, indomethacin reduced plasma levels of total bilirubin in cirrhotic rats, which was not exerted by selective COX inhibitors. This has not been identified previously except that indomethacin reduced plasma bilirubin levels in patients with acute obstructive cholecystitis [27]. Besides, in infants with patent ductus arteriosus, indomethacin lowered plasma bilirubin levels [28]. On the other hand, the renal toxicity of COX inhibitor has raised concerns in the cirrhotic patients [29]. However, our data revealed that 2-week selective or non-selective COX inhibitors treatment did not influence plasma levels of creatinine in cirrhotic rats. In addition, we previously found that acute administrations of selective COX-1 or COX-2 inhibitor did not alter the calculated creatinine clearance rates of cirrhotic rats [30]. Therefore, a short-term use of selective COX inhibitors in cirrhosis might be relatively safe in terms of renal function. Nevertheless, cautions should be paid case-by-case in clinical practice.

A previous investigation showed that a single dose of 50 mg indomethacin neither improved gas exchange nor affected systemic and pulmonary hemodynamics in patients with HPS [31]; however, a series of cases report showed that a long-term treatment of 40mg/kg/day aspirin, as well as a COX-1 inhibitor, could improve hypoxia and reduce intrapulmonary shunts in 3 children [32]. Our current data suggest that chronic indomethacin treatment is more efficacious than acute administration in alleviating hypoxia in animals with experimental HPS, although in order to avoid detrimental influences on hemodynamics and survival, the dose and duration in clinical setting deserve further clarification.

It is worth noting that SC-560 as well as celecoxib down-regulated both pulmonary COX-1 and COX-2 protein expressions in the current study. In fact, SC-560 is a preferential but not absolute COX-1 inhibitor (COX-1 IC_50_ = 0.009 mM; COX-2 IC_50_ = 6.3 mM) [33]. Likewise, celecoxib causes a preferential but not absolute COX-2 blockade. Therefore, both of them down-regulated COX-1 and COX-2 expressions with different magnitude, which made the statistical significance not remarkable between SC-560 and celecoxib.

Our data showed that non-selective COX inhibitor and selective COX-1, but not COX-2 inhibitor, decreased intrapulmonary shunts in cirrhotic rats. The attenuations of intrapulmonary shunts might be related to direct constriction of intrapulmonary vessels or inhibition of angiogenesis. Prostacyclin is a potent vasodilator affecting vascular tone in cirrhosis [6]. Regard the vascular reactivity, we found previously that selective COX-1 inhibition by SC-560 significantly reduced plasma prostacyclin levels; on the contrary, COX-2 inhibition by NS-398 only exerted modest effects [34]. Consistent with our findings, Bolego et al. have reported that COX-1, but not COX-2, played a critical role in prostacyclin production in human endothelial cell [35]. It is thus inferred that prostacyclin inhibitions by selective COX-1 inhibition may participate more than COX-2 inhibition in alleviating the degree of intrapulmonary shunts via vascular tone modification.

The selective COX inhibitors also diminished intrapulmonary vWF-positive staining cells accompanied by VEGF protein down-regulation, suggesting the effect of anti-angiogenesis. In line with our finding, Gately et al. reported that COX-2 inhibition blocked VEGF signaling pathway then suppressed angiogenesis [36]. Inhibition of COX-1 enzyme by SC-560 also decreased angiogenesis in human ovarian cancer xanografts by abolishing VEGF [37]. Our data showed that COX-2 inhibition also diminished pulmonary vWF-contained cells; however, the protein expressions of VEGFR-2, phosporylated Akt and ERK (42) were significantly up-regulated by celecoxib. The VEGFR-2/Akt/ERK is an important pathway in developing HPS [3,15]. Up-regulation of VEGFR-2/Akt/ERK by celecoxib might have neutralized its effect on anti-angiogenesis. Taken together, the failure of COX-2 inhibition to abolish intrapulmonary shunts may be ascribed to its weaker capacity to constrict intrapulmonary vessels and divergent effects on VEGF-mediated pathway.

Activation of NFκB is a key event of inflammation. The present study demonstrated that COX inhibition attenuated pulmonary NFκB and phosphorylated IκB protein expressions. Consistently, we have reported that rosuvastatin ameliorated HPS via NFκB signaling disruption [4]. It has been shown that aspirin can inhibit the activity of NFκB [38]. Pro-inflammatory stimuli also increase VEGF synthesis through the NFκB-dependent pathway [39]. Therefore, the inhibition of pulmonary NFκB expression participates, at least partly, in the COX inhibition-induced alleviation of pulmonary inflammation and angiogenesis.

In conclusion, our work demonstrates that a 2-week selective COX inhibition improves HPS in cirrhotic rats, in which anti-angiogenesis and anti-inflammation play roles and COX-1 inhibition is more promising.

## Supporting information

S1 Table Hemodynamic and biochemistry parameters of CBDL rats receiving vehicle (control) or indomethacin for 1 week. (PDF)
