# Supplementary material for: Selective cyclooxygenase inhibition by SC-560 improves hepatopulmonary syndrome in cirrhotic rats
Source: PLoS One. 2017 Jun 20;12(6):e0179809. doi: 10.1371/journal.pone.0179809 (PMC5478154; doi:10.1371/journal.pone.0179809)
Supplement: S1 Table — (PDF) [file pone.0179809.s001.pdf]

Supplementary table

Hemodynamic and biochemistry parameters of CBDL rats receiving vehicle (control) or indomethacin for 1 week

	Control (n=7)	Indomethacin (n=8)
Body weight (g)	354 ± 28	353 ± 6
MAP (mmHg)	107 ± 18	115 ± 11
PP (mmHg)	17.9 ± 2.0	16.7 ± 3.6
HR (beats/min)	366 ± 63	395 ± 43
ALT (IU/L)	183 ± 30	188 ± 37
AST (IU/L)	773 ± 134	1186 ± 526
TB (mg/dL)	8.2 ± 1.1	8.4 ± 1.1
Cr (mg/dL)	0.3 ± 0.15	0.3 ± 0.15

MAP: mean arterial pressure; PP: portal pressure; HR: heart rate; AST: aspartate aminotransferase; ALT: alanine aminotransferase; TB: total bilirubin; Cr: creatinine; $P>0.05$ between indomethacin-treated and control group.

- In order not to make the manuscript too lengthy, this table is not shown in the Revised manuscript.
